# NF‐κB signalling pathways in nucleus pulposus cell function and intervertebral disc degeneration

**DOI:** 10.1111/cpr.13057

**Published:** 2021-05-24

**Authors:** Guang‐Zhi Zhang, Ming‐Qiang Liu, Hai‐Wei Chen, Zuo‐Long Wu, Yi‐Cheng Gao, Zhan‐Jun Ma, Xue‐Gang He, Xue‐Wen Kang

**Affiliations:** ^1^ Department of Orthopedics Lanzhou University Second Hospital Lanzhou China; ^2^ The Second Clinical Medical College Lanzhou University Lanzhou China; ^3^ Key Laboratory of Orthopedics Disease of Gansu Province Lanzhou University Second Hospital Lanzhou China; ^4^ The International Cooperation Base of Gansu Province for the Pain Research in Spinal Disorders Lanzhou China

**Keywords:** intervertebral disc degeneration, nuclear factor‐κB, nucleus pulposus

## Abstract

Intervertebral disc degeneration (IDD) is a common clinical degenerative disease of the spine. A series of factors, such as inflammation, oxidative stress and mechanical stress, promote degradation of the extracellular matrix (ECM) of the intervertebral discs (IVD), leading to dysfunction and structural destruction of the IVD. Nuclear factor‐κB (NF‐κB) transcription factor has long been regarded as a pathogenic factor of IDD. Therefore, NF‐κB may be an ideal therapeutic target for IDD. As NF‐κB is a multifunctional functional transcription factor with roles in a variety of biological processes, a comprehensive understanding of the function and regulatory mechanism of NF‐κB in IDD pathology will be useful for the development of targeted therapeutic strategies for IDD, which can prevent the progression of IDD and reduce potential risks. This review discusses the role of the NF‐κB signalling pathway in the nucleus pulposus (NP) in the process of IDD to understand pathological NP degeneration further and provide potential therapeutic targets that may interfere with NF‐κB signalling for IDD therapy.

## Graphical abstract

Intervertebral disc degeneration (IDD) is a common clinical degenerative disease of the spine. A series of factors, such as inflammation, oxidative stress, and mechanical stress, promote degradation of the extracellular matrix (ECM) of the intervertebral discs (IVD), leading to dysfunction and structural destruction of the IVD. Nuclear factor‐κB (NF‐κB) transcription factor has long been regarded as the pathogenic factor of IDD. Therefore, NF‐κB may be an ideal therapeutic target for IDD. As NF‐κB is a multifunctional functional transcription factor with roles in a variety of biological processes, a comprehensive understanding of the function and regulatory mechanism of NF‐κB in IDD pathology will be useful for the development of targeted therapeutic strategies for IDD, which can prevent the progression of IDD and reduce potential risks. This review discusses the role of the NF‐κB signalling pathway in the nucleus pulposus (NP) in the process of IDD to understand pathological NP degeneration further and provide potential therapeutic targets that may interfere with NF‐κB signalling for IDD therapy.

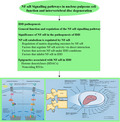

## 1 INTRODUCTION

Intervertebral disc degeneration (IDD) is a common degenerative disease of the musculoskeletal system and is also the main cause of chronic low back pain (LBP), which seriously affects the quality of life of patients and places a huge economic burden on families and society.^1^, ^2^, ^3^ It is estimated that about 20% of adolescents have mild IDD and 80% of the general population will experience back pain symptoms during their lifetime.^4^ However, the specific pathogenesis of IDD is still not fully understood. At present, IDD is believed to be a complex cell‐mediated process that ultimately leads to changes in intervertebral disc (IVD) structure and function.^5^

As the joint connects the vertebral body, the IVD is the most critical part of the spine weight‐bearing system and is the tissue that undergoes degenerative changes earliest in the human body.^6^ A healthy IVD is composed of a central gel‐like nucleus pulposus (NP), an annulus fibrosus (AF) surrounding the NP, and two piece of cartilage endplate (CEP) connecting the upper and lower vertebral bodies. They play important roles in maintaining the stability of the spine.^7^ Current evidence shows that a series of factors, including inflammation, oxidative stress, mechanical stress, etc, promote the degradation of IVD extracellular matrix (ECM), leading to IVD dysfunction and structural destruction.^8^, ^9^, ^10^ NF‐κB signalling is very important for maintenance of IVD homeostasis.^11^, ^12^, ^13^, ^14^ Based on the available literature, we highlight the NF‐κB signalling pathway and changes in NF‐κB expression, multiple biological functions, as well as the therapeutic potential of signalling for IDD, to obtain a better understanding of the crucial role of NF‐κB signalling in IDD.

## 2 IDD PATHOGENESIS

IDD is not a single spine disease, but rather, a common complex disorder associated with multiple risk factors that contribute to the progression of IDD. Various internal and external factors, including genetic susceptibility, mechanical stress, heavy workload, smoking, obesity and ageing, can lead to the development of IDD, such as IVD structural instability, intervertebral space collapse, intervertebral disc herniation, cartilage calcification, spinal stenosis and radiculopathy.^15^, ^16^, ^17^, ^18^, ^19^ Multiple studies have shown that pathological factors such as ECM degradation, inflammatory response, oxidative stress, mitochondrial dysfunction, telomere shortening and DNA damage, nutritional deprivation, abnormal mechanical load and epigenetic changes participate in the progression of IDD.^4^, ^20^, ^21^, ^22^, ^23^, ^24^

### 2.1 ECM degradation

The physiological function of IVD depends on the molecular composition of the NP ECM. The NP is composed of ECM rich in type II collagen, elastin and proteoglycan, and it acts to offset and transmit the axial pressure load of the spine during a stress process.^20^, ^25^ The primary function of AF, which is composed of alternating type I collagen fibres, is to prevent the NP from protruding under pressure during the bending or twisting of the spine. CEP is a uniformly thick hyaline cartilage tissue, and its ECM is primarily composed of proteoglycans and collagen fibres. Because IVD is an avascular and nerveless tissue, CEP plays an important role in supplying nutrients to the intervertebral disc. The IVD exchanges nutrients and metabolic waste by diffusion through the CEP, thereby maintaining its normal structure and function.^26^ Due to NP tissue not only lacks blood vessels and nerves, but also has limited repair ability after injury, so the protection of NP cells is very important for maintaining IVD health.^27^ NP cells maintain homeostasis by synthesizing ECM, thereby retaining its structural and functional integrity. However, during the progression of IDD, NP cells lose the ability to maintain their integrity and survivability. This leads to the gradual loss of type II collagen and proteoglycan, resulting in a reduction in the IVD height, loss of the boundary between AF and NP and a decreased ability to withstand mechanical loads.^20^, ^25^ Matrix metalloproteinases (MMPs) and a disintegrin and metalloproteinase with thrombospondin motifs (ADAMTSs) are the main catabolic enzymes in the NP and include MMP‐3, MMP‐9, MMP‐13, ADAMTS‐4 and ADAMTS‐5. The expression of metabolic enzymes is increased in degenerated NP tissue; therefore, the catabolism of NP cells is greater than the protective function of NP cell anabolism, which eventually leads to IDD.^28^, ^29^

### 2.2 Inflammation

As IDD progresses, the levels of proinflammatory factors, such as IL‐1α, IL‐1β, IL‐6, IL‐8, IL‐17 and TNF‐α, increase significantly. These factors produce inflammatory stimuli on the sinus vertebral nerve endings resulting in nerve root pain, which is the main cause of chronic LBP in patients.^4^ IL‐1β is the most intensively studied proinflammatory factor. It has been shown to trigger higher levels of proinflammatory mediators (including TNF‐α, IL‐6 and several matrix‐degrading enzymes), disrupt the balance of ECM metabolism and impair its metabolism.^30^ TNF‐α and IL‐6 are essential proinflammatory factors that have been shown to be closely related to the progression of IDD.^31^, ^32^ TNF‐α can trigger inflammation, cause nerve swelling and neuropathic pain and aggravate cell apoptosis due to its cytotoxic effect, in individuals with LBP.^33^ Krupkova et al ^34^ showed that IL‐1β aggravated the local inflammatory response of IVD cells by activating the NF‐κB pathway and aggravating nerve radiation pain in rats.

### 2.3 Oxidative stress

With the progression of IDD, the levels of reactive oxygen species (ROS) in IVD increase significantly, including those of superoxide anions (O^2‐^), hydroxyl radicals (OH), hydrogen peroxide (H_2_O_2_) and nitric oxide (NO), which are the by‐products of cellular oxidative metabolism.^35^ NP cells are the most important functional cells in the IVD. They have been shown to be non‐anaerobic and carry out aerobic metabolism in the body, with ROS being the main metabolic by‐product.^36^ Excessive ROS‐mediated oxidative stress accelerates the IDD process through NF‐κB signalling pathway.^37^ Feng et al ^38^ cultured rat NP cells under conditions of 20% O_2_ or 1% O_2_. Overproduction of ROS under conditions of 20% O_2_ was shown to induce NP cell catabolism and increase expression of inflammatory factors through the NF‐κB signalling pathway.

### 2.4 Mitochondrial dysfunction

Mitochondria contain important components that complete the tricarboxylic acid cycle and redox reactions, which lead to the production of adenosine triphosphate (ATP) through oxidative phosphorylation, thereby ensuring normal cell function.^39^ Mitochondrial DNA damage intensifies with age, leading to mitochondrial dysfunction and abnormal electron leakage, thereby increasing ROS production. At the same time, the reduction of cellular redox balance leads to increased oxidative damage to cells by ROS, which in turn mediates cell damage. The impaired mitochondrial function in IVD cells is involved in the occurrence and development of IDD.^40^ Studies have shown that the accumulation of the progerin protein in rat NP cells can increase the levels of ROS, destroy the mitochondrial membrane potential, reduce ATP production and change the activity of mitochondrial enzyme complexes, thereby destroying the structure and function of mitochondria and accelerating the IDD process.^41^ Chen et al ^42^ studied the effect of bone marrow mesenchymal stem cells (BMSCs) on the regeneration of NP cells and found that co‐culture with BMSCs can reduce the levels of ROS in NP cells and maintain the cell mitochondrial membrane potential and mitochondrial integrity, thereby reducing stress‐induced mitochondrial damage in NP cells. Interestingly, excessive ROS‐mediated oxidative stress further accelerates the IDD process through the NF‐κB signalling pathway.^37^ Accordingly, mitochondrial dysfunction leads to the accumulation of intracellular ROS which plays an important role in the IDD process.

### 2.5 Telomere shortening and DNA damage

Mammalian telomeres are composed of repetitive DNA fragments (TTAGGG) that are 50‐400 nucleotides in length.^43^ Due to incomplete replication of DNA ends, telomere length gradually shortens over multiple rounds of cell division. Studies have shown that with the progression of IDD, telomere length gradually shortens and telomerase activity gradually decreases. Jeong et al ^44^ extracted and cultured human NP cells from patients of different ages (35, 42, 55, 66 and 76 years) undergoing IVD surgery and found that as the cells replicated, the NP cells showed different degrees of ageing characteristics, specifically an increase in the number of SA‐β‐gal‐positive cells, shortened telomeres and decreased telomerase activity. Wu et al^45^ used a lentiviral vector to transfer human telomerase reverse transcriptase (hTERT) into senescent human NP cells and found that as the cells replicated, the telomere length was maintained and the telomerase activity recovered, cell senescence was delayed, and the proliferation rate of NP cells increased. DNA damage refers to physical or chemical factors that cause damage to DNA double strands in cells, leading to gene mutations and chromosomal rearrangements. In severe cases, it can lead to the loss of genetic information, cell cycle arrest and apoptosis.^46^ Studies have shown that telomere shortening often causes DNA damage in cells and is an internal trigger for cell senescence.^21^ Experimental results have shown that ionizing radiation can cause intracellular DNA damage and increase the number of p16‐positive cells in NP tissues of wild‐type mice.^47^ Nasto et al^48^ exposed adult wild‐type and DNA repair gene Ercc1‐deficient mice to ionizing radiation to induce DNA damage and studied its effect on the IVD structure. They found that in the IVD of the Ercc1‐deficient mice, NP cell senescence and apoptosis increased significantly. Importantly, telomere shortening and DNA damage aggravate cell damage by activating the NF‐κB signalling pathway to further increase the release of proinflammatory cytokines, thereby accelerating cellular senescence.^49^

### 2.6 Nutrient deprivation

A reduction in cell nutrition is another important cause of IDD. Previous studies have shown that serum starvation can inhibit the proliferation of artificially cultured IVD cells and increase their senescence rate, while administration of high concentrations of serum can increase the proliferation rate of NP cells.^50^ A variety of growth factors derived from serum can promote cell proliferation, including insulin‐like growth factor (IGF), fibroblast growth factor (FGF), platelet‐derived growth factor (PDGF) and transforming growth factor‐β (TGF‐β).^50^, ^51^ It is worth noting that in the pathological process of CEP degradation, the apoptosis and calcification of CEP cells block the nutrient supply of NP cells, thereby accelerating the senescence and apoptosis, ultimately leading to IDD.^21^ In addition, aggravated NP degeneration is associated with a decreased supply of nutrients. Under the condition of serum deprivation, the NF‐κB signalling pathway is activated, which further aggravates the apoptosis of NP cells.^52^

### 2.7 Abnormal mechanical load

The human spine is subjected to multi‐directional mechanical loads, including axial, radial, and circumferential compression, tension and shear. Abnormal mechanical load is a risk factor for IDD.^21^ Studies have shown that an increase in the mechanical load on the spine caused by obesity is a risk factor for the development of IDD. The increase in body mass index changes the biomechanics of IVD and ultimately leads to the narrowing of the intervertebral disc space and the enhancement of the catabolism of the IVD cells.^53^ Liang et al^54^ used forelimb amputation to induce rats to walk upright to simulate the upright posture of humans and found that a long‐term upright posture can cause NP tissue structure disorder, AF fissures, IVD height reduction and ECM decomposition were enhanced. Using the known hydrophobicity of the experimental mice, Ao et al^22^ placed the experimental group of mice in a space with 5‐mm deep water to induce their bipedal standing posture (6 hours per day), while the control group was kept under normal conditions for 10 weeks. They found that IDD in the experimental group was significantly worsened. In addition, NP cells are known to convert mechanical load into biological signals, which can affect cell survival or death by regulating gene transcription, and abnormal mechanical load can increase NP cell catabolism through the NF‐κB signalling pathway, while decrease anabolism.^14^, ^21^, ^53^

### 2.8 Epigenetic changes

Epigenetics refers to reversible and heritable changes in gene function that do not involve alterations to the DNA sequences. It mainly includes DNA methylation, histone modification and non‐coding RNA (ncRNA). These changes regulate the specific expression of genes through the interaction between the environment and the genome to make permanent changes and play an important role in the occurrence and development of diseases.^55^ Ikuno et al^23^ showed that in the late stage of IDD, the degree of DNA methylation increased significantly. Yang et al^24^ reported that miR‐143‐5p was highly expressed in degenerated NP cells, and after adding miR‐143‐5p‐specific inhibitors to NP cells, cell proliferation increased and apoptosis decreased. Epigenetic changes can further affect the IDD process by regulating the NF‐κB signalling pathway.^8^ Unfortunately, the research on epigenetic changes in IDD is still in its infancy, and the specific mechanisms still need to be clarified.

## 3 GENERAL FUNCTION AND REGULATION OF THE NF‐κB SIGNALLING PATHWAY

**FIGURE 1:**
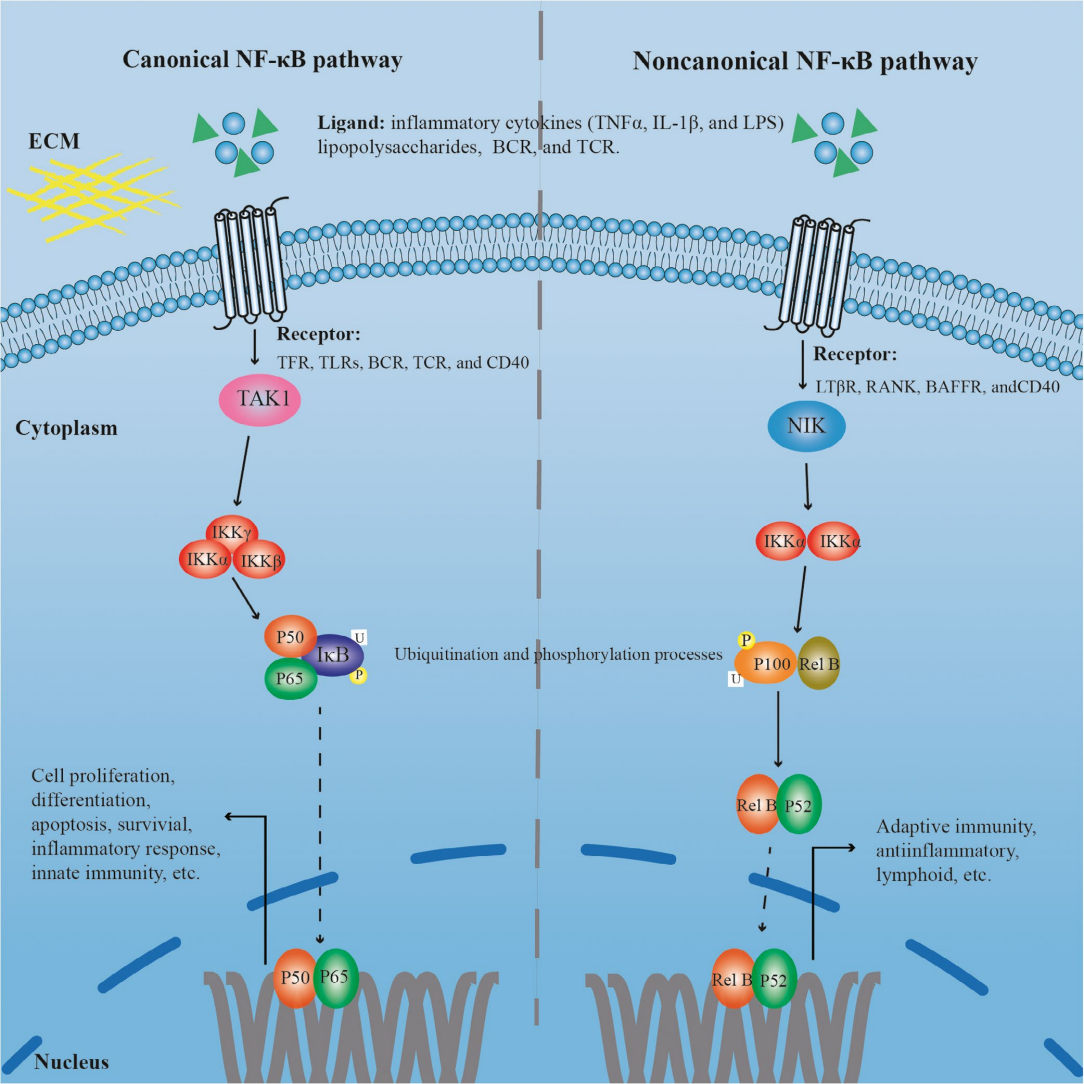
Canonical and non‐canonical NF‐κB signalling pathways

NF‐κB, first discovered in 1986 by Sen et al,^56^ is a transcription factor that binds specifically to the enhancer region of the immunoglobulin κ light chain gene.^57^ It was originally thought to be a regulator of B cell differentiation and function, but was later shown to be a ubiquitous transcription factor expressed in various cells. It plays a key regulatory role in immune response, inflammatory response and tumourigenesis. NF‐κB exists as a dimer, which is a member of the Rel protein family that share a conserved sequence of about 300 amino acids at the N‐terminus known as the Rel homology domain (RHD), which is derived from the retroviral oncoprotein, v‐Rel, and is involved in DNA binding and dimerization, and is related to inhibitor of κB (IκB) proteins.^58^ Common Rel proteins are Rel A (p65), Rel B, c‐Rel, p50 and p52. The five Rel family proteins can be divided into two categories. The first category includes p50 and p52, which are produced from the precursor proteins p105 (NF‐κB1) and p100 (NF‐κB2), respectively.^59^, ^60^ The second category includes p65, Rel B and c‐Rel, which are processed into mature proteins with transcription transactivation domains (TADs).^61^, ^62^ Although other combinations, including homodimers p50:p50 and p52:p52 or heterodimers p52:Rel A and p50:Rel B, participate in different functions, two typical dimers (p50:p65 and p52:Rel B) are the most widespread in eukaryotic cells and have the most important regulatory roles^62^ (Figure 1).

Although there are many different ways of activating NF‐κB, two major signalling pathways have been reported to lead to the activation of NF‐κB target genes. These are referred to as the canonical and non‐canonical pathways, in which the canonical approach dominates.^63^ Generally, the activation of NF‐κB can be summarized as the transfer of stimulating signals from ligands, receptors, adaptor proteins and deoxycytidine kinase (dCK) complexes to NF‐κB.^62^, ^64^ Among them, the tumour necrosis factor receptor 1 (TNFR1)‐, interleukin 1 receptor (IL‐1R)‐, Toll‐like receptor (TLR)‐, T cell receptor (TCR)‐ and B cell receptor (BCR)‐mediated NF ‐κB activation pathways have been studied most extensively.^44^ In the resting state, NF‐κB binds to IκB, a specific inhibitor of NF‐κB, and exists in the cytoplasm as an inactive complex. It binds to the RHD region of NF‐κB to prevent it from entering the nucleus to perform its function. The IκB proteins mainly include IKKα (IKK1), IKKβ (IKK2) and regulatory subunit IKKγ. IKKγ forms an IKK complex with IKKα/IKKβ dimers.^56^ When cells are stimulated by inflammatory factors, oxidative stress, mechanical stress and biochemical toxins, the IKK complex is activated by the upstream activator TGFβ‐activated kinase 1 (TAK1) interacting with IKKγ and, then, under the action of ubiquitin ligase, the molecular conformation of IκB changes, which is recognized and degraded by the 26S proteasome, resulting in the activation of NF‐κB. IκB is dissociated from the trimer, exposing the translocation signal on the p50 subunit and the DNA binding site on the p65 subunit, thereby activating NF‐κB and translocating from the cytoplasm to the nucleus, and interacting with the κB group. This complex regulates the transcription of many target genes related to cell proliferation, differentiation, apoptosis, inflammation and proangiogenic functions, leading to the production and release of inflammatory cytokines, such as TNF‐α, IL‐2, IL‐6 and INF‐γ. In turn, the released cytokines can reactivate NF‐κB, which can mediate tissue inflammatory damage through a cascade reaction^65^ (Figure 1).

The non‐canonical NF‐κB activation pathway is mediated by the transcription factor p100/Rel B, which is mainly related to immune cells. The non‐canonical NF‐κB pathway is activated by a select group of TNFR superfamily members, including CD40, the lymphotoxin‐β receptor (LTβR) and the receptor activator of NF‐κB (RANK). NF‐κB‐inducing kinase (NIK, also known as MAP3K14) and IKKα are necessary for the processing of p52 from p100 and result in dimerization and activation of the p52/Rel B heterodimer.^66^ The activation of the non‐canonical pathway of the NF‐κB signalling pathway mainly depends on IκBα, rather than IκBβ and IκBγ. In the resting state, the N‐terminus of p100 exhibits self‐inhibition, blocking the transcriptional activity of p100/Rel B. Ligand stimulation, such as the binding of CD40L and CD40, can eliminate K48 ubiquitination of the key protein NIK (NF‐κB). κB induces kinase and stops the degradation of NIK by the proteasome. Stable NIK activates p100, which leads to phosphorylation and ubiquitination of p100, which is recognized by the proteasome and partially degraded into p52. Finally, the nuclear translocation of p52 and Rel B activates transcription of target genes^57^, ^66^ (Figure 1).

## 4 SIGNIFICANCE OF NF‐κB IN THE PATHOGENESIS OF IDD

The destruction of the NP ECM is due to a decrease in NP cell anabolism and increase in catabolism/apoptosis. Genes that either increase or decrease the susceptibility to IDD have been identified using several models of IDD. The NF‐κB pathway is one of the better characterized signalling pathways activated by IDD stimuli, such as inflammation and mechanical loading. Studies using loss‐of‐function approaches confirmed the importance of NF‐κB in IDD. In cultured NP cells, the NF‐κB‐specific inhibitor, BAY11‐7082, significantly inhibited IL‐1β‐induced catabolic gene expression, such as MMP‐3, MMP‐9, MMP‐13, ADAMTS‐4 and ADAMTS‐5.^67^ In the NP degeneration model, NF‐κBp65 can be knocked down in IVD by injection of specific siRNA, thereby reducing degeneration of the NP caused by injury.^68^ Not surprisingly, IKKs, as upstream regulators of the mechanism of NF‐κB activation, are also involved in the catabolism and degeneration of NP cells. In a DNA repair‐deficient mouse model of accelerated ageing (Ercc1‐/Δ mice), intraperitoneal injection of 8K‐NBD peptide, which inhibits the activation of NF‐κB by inhibiting the formation of IKK protein complexes, has a positive effect on NF‐κB. After activation for systemic inhibition, it reduces age‐related IDD, including increased proteoglycan synthesis, and ameliorates loss of IVD cellularity and matrix proteoglycan.^69^

## 5 NP CELL CATABOLISM IS REGULATED BY NF‐κB

### 5.1 Regulation of matrix‐degrading enzymes by NF‐κB

Understanding the molecular mechanism underlying the initiation of NP catabolism after activation of the NF‐κB signalling pathway will help to improve understanding of potential therapeutic targets for IDD. NF‐κB directly or indirectly induces the expression of matrix‐degrading enzymes and other IDD‐related factors, thereby coordinating abnormal NP catabolic pathways. NF‐κB induces the expression of catabolic genes through the NF‐κB response elements located in the promoters of MMP‐1, MMP‐3, MMP‐9, MMP‐13 and ADAMTS‐5 genes.^70^, ^71^, ^72^ In addition, NF‐κB signalling pathway promotes the expression of chemokines CCL3 and CCL4 in IDD and further aggravates the inflammatory response.^73^ Importantly, activation of the NF‐κB pathway promotes the expression of inflammatory mediators, including inducible nitric oxide synthase (iNOS) and cyclooxygenase (COX‐2) genes. iNOS and COX‐2 produce nitric oxide (NO) and induce prostaglandin (PGE2) gene expression. They are not only cell signalling molecules but also well‐known pathogenic factors for IDD. NO and PGE2 can inhibit the synthesis of aggrecan in NP, leading to the destruction of the structure and function of the extracellular matrix.^74^ In addition, increased PGE2 level can affect the catabolic/anabolic balance in the IVD, for example by promoting the expression of MMPs to further accelerate IVD degeneration.^75^ NF‐κB can also upregulate other transcription factors, such as hypoxia‐inducible factor 2α (HIF‐2α), which is regulated in development, and DNA damage response 1 (REDD1) and prolyl hydroxylase 3 (PHD3). Activated HIF‐2α binds to the HIF‐2α binding motif of the promoter of target genes and promotes the expression of matrix‐degrading enzymes, such as MMP‐13 and ADAMTS‐4. HIF‐2α controls the catabolic factors, MMP‐13 and ADAMTS‐4, which regulate metabolism of type II collagen and aggrecan.^76^ REDD1, also known as RTP801, DDIT4, or DIG2, was initially identified as a hypoxia‐inducible factor 1 (HIF1) response protein, and the level of REDD1 expression was shown to be markedly increased under hypoxic conditions.^77^ As a transcription factor, NF‐κB binds to the REDD1 promoter region, and hypoxia can protect against ECM degradation caused by serum deprivation through the NF‐κB/REDD1 pathway.^52^ PHD3 is another important molecule involved in HIF‐1α signalling, which forms a regulatory circuit with NF‐κB and acts as a transcription coactivator. NF‐κB‐dependent induction of ADAMTS‐5, MMP‐13 and COX‐2 is significantly reduced by PHD3 loss of function.^78^ Taken together, these findings clearly indicate that the above factors are key components of the NF‐κB signal transduction pathway in NP cells, which are likely to play key roles in the pathogenesis of IDD and represent new pharmacological targets.

### 5.2 Factors that regulate NF‐κB activity via direct interaction

**FIGURE 2:**
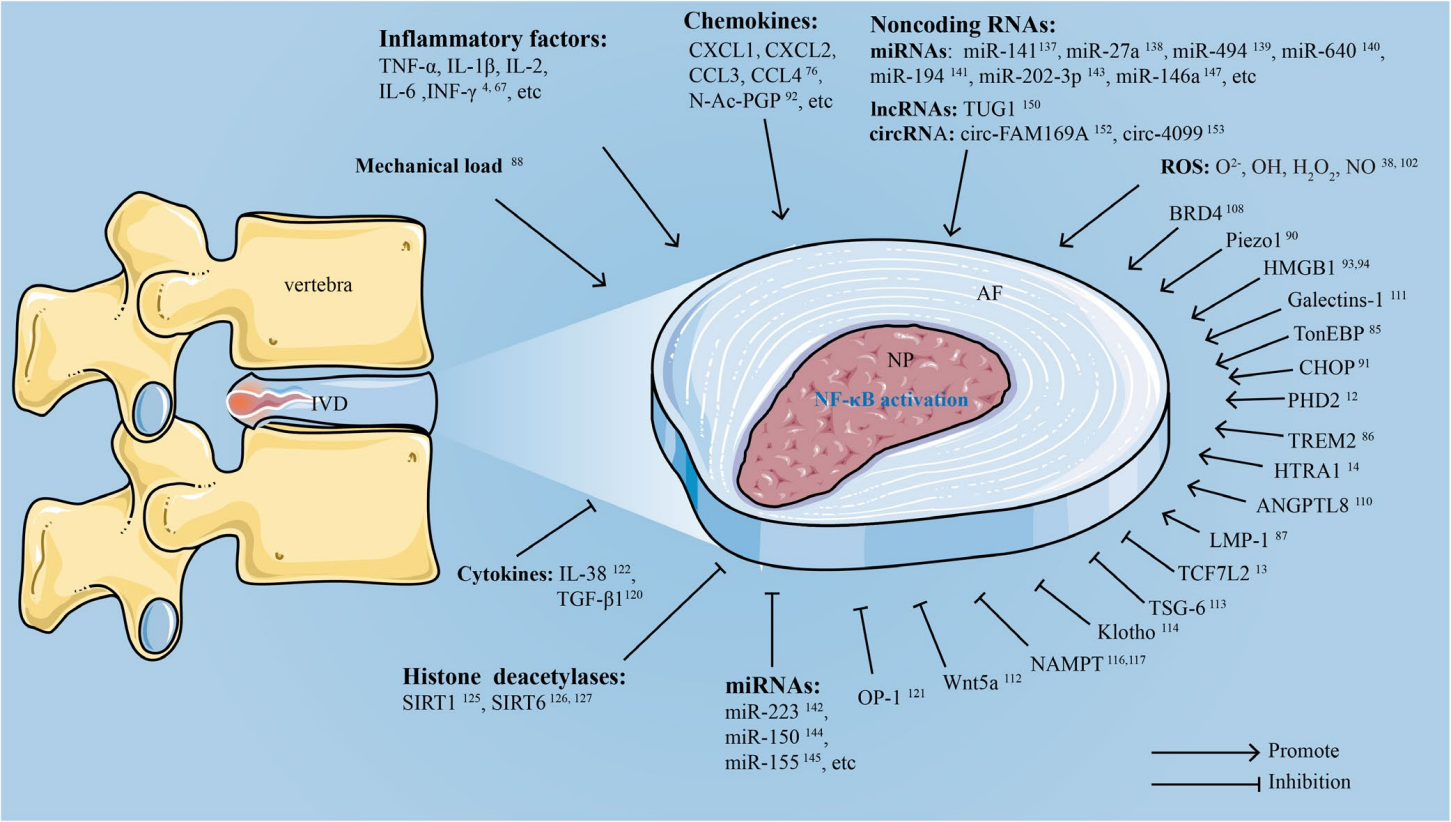
Genes or factors that positively or negatively regulate NF‐κB activation in NP

The catabolism of NF‐κB in NP cells is enhanced by several NF‐κB binding proteins. Figure 2 shows the genes that have a stimulatory or inhibitory effect during the activation of NF‐κB in NP cells. Stimulators that bind to the NF‐κB subunit include tonicity‐responsive enhancer‐binding protein (TonEBP), triggering receptor expressed on myeloid cells‐2 (TREM2), prolyl‐4‐hydroxylase domain protein 2 (PHD2), transcription factor 7‐like 2 (TCF7L2) and LIM mineralization protein‐1 (LMP‐1). Previous studies have shown that TonEBP is necessary for the survival and function of NP cells in the osmotically challenging environment of IVD.^79^, ^80^ TonEBP activates LPS‐induced NF‐κB activity by recruiting the transcriptional coactivator p300 to the NF‐κB enhancer and regulates the expression of CXCL1, CXCL2 and CXCL3, thereby inducing the inflammatory environment of NP cells.^81^ TREM2 is a cell surface receptor that belongs to the lectin‐like immunoglobulin superfamily. TREM2 acts as a promoter in the degeneration of human NP cells.^82^ The expression of TREM2 is increased in degenerated NP cells, promotes NP cell apoptosis and increases the secretion of inflammatory factors (TNF‐α, IL‐1β and IL‐6) by promoting nuclear translocation of NF‐κBp65, while cell proliferation is weakened. After downregulating the expression of TREM2 in human degenerated NP cells transfected with TREM2‐siRNA, the nuclear translocation of NF‐κBp65 was significantly inhibited.^82^ PHD2 is a member of the 2‐oxoglutarate/Fe^2+^‐dependent dioxygenase superfamily. PHD2 in NP cells binds directly to NF‐κBp65 affecting its transcriptional activity, thereby inducing the expression of inflammatory factors TNF‐α and IL‐1β and the activation of NF‐κB.^11^ TCF7L2 is a key transcription factor in the canonical Wnt pathway and plays a vital role in the degradation of cell matrix. Loss‐of‐function experiments using TCF7L2‐siRNA or lentiviral shTCF7L2 showed that in the absence and presence of TNF‐α, TCF7L2 knock‐down can inhibit NF‐κB signalling and matrix degradation by inhibiting the activity of the p65/RelA promoter.^12^ LMP‐1 has been shown to induce sulphated glycosaminoglycan (sGAG) production in NP. LMP‐1 eliminates TNF‐α‐induced MMP‐3 and MMP‐13 expression by inhibiting p65 translocation and MMP‐3 and MMP‐13 promoter activity.^83^ Therefore, targeting these factors that affect NF‐κB signalling may represent a new therapeutic strategy for IDD.

### 5.3 Factors that activate NF‐κB under IDD conditions

Previous studies have shown that mechanical load is one of the risk factors for IDD. IVD cells are known to convert mechanical stress into biological signals, which can be integrated into cell responses and affect cell survival or death by regulating gene transcription. Moderate mechanical load has a protective effect on NP cells, while abnormal mechanical load can increase NP cell catabolism through the NF‐κB signalling pathway, while decrease anabolism. For example, Zhang et al ^84^ established an in vitro mechanical load model of human NP cells. They reported that a short mechanical load (4 hours) followed by a long rest (20 hours) had a protective effect on the matrix degradation of NP cells. However, after a longer loading time (20 hours), a shorter rest time (4 hours) activates the activation of the NF‐κB signalling pathway and ultimately leads to increased NP cell apoptosis and ECM degradation. Piezo1 is a new mechanosensitive cation channel encoded by the FAM38A gene, which allows the non‐selective passage of divalent ions Ca^2+^, Mg^2+^ and Mn^2+^.^85^ Previous studies have shown that Piezo1 is a transmembrane ion channel. The triangular pyramid structure outside the cell membrane is responsible for sensing mechanical signals and transmitting them to the porous core structure of Piezo1, and then converting the mechanical signals into chemical signals. Abnormal mechanical load can upregulate the level of IL‐1β expression in NP cells through Piezo1, which in turn activates the downstream NF‐κB signalling pathway leading to the inflammatory microenvironment of NP tissue and acceleration of the IDD process.^86^ In summary, although moderate mechanical load has a beneficial effect on NP cells, excessive mechanical load inhibits the viability of human NP cells and promotes ECM degradation and an inflammatory microenvironment by activating the NF‐κB signalling pathway. Therefore, research regarding how mechanical load affects IVD metabolism may be crucial to the development of innovative IDD prevention and treatment strategies (Figure 2).

It is well known that inflammatory cytokines cause the induction of catabolic genes in NP cells through a mechanism involving activation of the NF‐κB signalling pathway. Therefore, treatment of NP cells with traditional inflammatory cytokines, such as IL‐1β and TNF‐α, has been widely used to prepare in vitro IDD models. IL‐6 is a well‐known NF‐κB target gene, and it also has a pathogenic effect in the progression of IDD.^4^ TNF‐α induces an increase in the expression of CHOP in NP cells and promotes NP cell apoptosis through NF‐κB signalling.^87^ N‐Acetylated proline‐glycine‐proline (N‐Ac‐PGP) is a chemokine involved in inflammatory diseases, and its expression is increased in degenerated IVDs. N‐Ac‐PGP has been shown to activate the NF‐κB signalling pathway in NP cells, thereby promoting the expression of proinflammatory factors and matrix catabolic enzymes.^88^ Another inflammatory mediator, high mobility group box 1 (HMGB1), is a chromatin protein with dual functions as a nuclear factor and an extracellular factor. It can also activate the NF‐κB signalling pathway in NP cells. Recently, HMGB1 has been recognized as an effective proinflammatory mediator in the degenerative IVDs.^89^ In the rat lipopolysaccharide (LPS)‐induced IDD model, HMGB1 is significantly upregulated in the IVDs. HMGB1 promotes the release of inflammatory cytokines in human IVD cells through the NF‐κB signalling pathway.^90^ Accumulating evidence shows that endogenous molecules produced by cellular metabolism or ECM degradation can trigger inflammatory responses, acting as ‘danger signals’ called damage‐associated molecular patterns (DAMPs).^91^ As a class of DAMPs, the accumulation of advanced glycation end products (AGEs) in NP tissues may activate the NF‐κB pathway, enhance the activity of NLRP3 inflammasomes and accelerate NP degeneration^92^ (Figure 2).

Due to the avascular nature of NP tissue, nutrient and metabolite transport are dependent on the osmotic effects of the CEP. Studies have shown that the oxygen content in NP is 1%, but NP cells are in a low‐oxygen but not completely anaerobic microenvironment.^93^, ^94^ NP cells are well adapted to the hypoxic microenvironment and have a certain level of oxidative metabolism.^95^, ^96^ With the progress of IDD, the oxygen concentration of NP tissue decreases further, and anaerobic glycolysis provides the main energy source accompanied by the accumulation of a large number of acidic metabolites, which then activate the NF‐κB signalling pathway.^38^ The acid‐sensing ion channels (ASICc) represent a subfamily of protonation channels, consisting of six isoforms (ASIC1a, 1b, 2a, 2b, 3 and 4), which was activated by extracellular acidosis, lactic acid and arachidonic acid. The extracellular lactic acid regulates intercellular ROS levels through ASIC1 and ASIC3, and the ROS further activate the NF‐κB signalling pathway, thereby promoting the activation of NLRP3 inflammasomes and the release of IL‐1β, both of which promote NP degeneration.^97^ In addition, under hypoxic conditions, IL‐1β promotes the catabolism of human NP cells through the NF‐κB signalling pathway.^98^ Other studies have shown that as IDD progresses, IVD tissue neovascularization increases, thereby exposing NP cells to higher oxygen tension.^36^, ^38^, ^99^ (Figure 2).

In addition, the abnormal expression of some genes in NP cells can further induce the development of IDD through the NF‐κB signalling pathway. High temperature requirement serine protease A1 (HTRA1) is a conserved PDZ serine protease, which belongs to the HTRA family of serine proteases. It is a secreted enzyme and is widely expressed in human cells and tissues.^100^ The degenerative changes of NP are known to be related to ECM degradation caused by locally produced MMPs and aggrecanase belonging to the ADAMTS family, resulting in tissue stiffness, loss of structural integrity and functional impairment. HTRA1 can induce the expression of ADAMTS‐5 in human NP cells through the ERK/NF‐κB/JNK signalling pathway.^13^ BRD4 expression is increased in degenerated NP tissues. BRD4 enhances NF‐κB signalling and activates autophagy to promote MMP‐13 expression in IDD.^70^ One of the members of the angiopoietin‐like (ANGPTL) protein family, angiopoietin‐like protein 8 (ANGPTL8), which is also known as betatrophin or lipasin, is a regulator of plasma lipid metabolism.^101^ The level of ANGPTL8 expression is increased in degenerated NP tissues. TNF‐α treatment has been shown to promote the expression of ANGPTL8 in NP cells in vitro. ANGPTL8 regulates ECM metabolism and inflammatory response by activating the NF‐κB signalling pathway.^71^ Galectin‐1 (Gal‐1), a member of the galectin family, may further accelerate IDD through NF‐κB signalling via enzymes that induce inflammatory mediators and ECM catabolism, such as IL‐6, CXCL8 and MMP‐1/3/13^102^ (Figure 2).

### 5.4 Factors that inhibit NF‐κB in IDD

The severity of IDD is often reduced when NF‐κB activity is restrained by factors required to maintain NP homeostasis. These factors that reduce NF‐κB activity in NP cells include Wnt5a, TNF‐α‐stimulated gene 6 protein (TSG‐6), Klotho, nicotinamide phosphoribosyltransferase (NAMPT), osteogenic protein‐1 (OP‐1) and IL‐38. Specifically, Wnt5a is a representative ligand that activates the β‐catenin independent pathway in Wnt signalling, and inhibits TNF‐α‐induced NF‐κB signalling, thereby reducing the expression levels of downstream catabolic genes. In the rat IDD model, the synthesis of type II collagen and aggrecan was reported to be increased after intradiscal injection of Wnt5a.^103^ TSG‐6 is a 30‐kD glycoprotein that can exert anti‐inflammatory effects. By inhibiting activation of the NF‐κB pathway, TSG‐6 can reduce the expression of MMP‐3 and MMP‐13 in IL‐1β‐treated NP cells and increase the expression of type II collagen and proteoglycan.^104^ The anti‐ageing protein, Klotho, has impressive anti‐inflammatory properties, and its expression is decreased in the degenerated NP. Overexpression of Klotho reduces the expression of inflammatory factors by inhibiting NF‐κB signalling.^105^ This makes increasing Klotho expression an attractive strategy for the treatment of IVD inflammatory diseases. NAMPT, also known as visfatin, is synthesized and secreted by fat, liver, skeletal muscle and other tissues.^106^ The NAMPT inhibitor, APO866, can reverse the matrix degradation induced by IL‐1β in NP cells through autophagy.^107^ Huang et al^108^ showed that the downregulation of NAMPT controls TNF‐α‐induced NLRP3 inflammasome activity and NP matrix degradation by inhibiting NF‐κB signalling. TGF‐β1 has been shown to promote the proliferation of NP cells, stimulate ECM synthesis, reduce the expression of ADAMTS and increase the expression of tissue metalloproteinase (TIMP), and TIMP is an inhibitor of MMP and ADAMTS.^73^, ^109^ TGF‐β1 exerts an anabolic effect on the intervertebral disc by inhibiting the NF‐κB pathway.^110^ OP‐1 is a growth factor that belongs to the transforming growth factor‐β (TGF‐β) family. OP‐1 effectively reduces NP cell apoptosis caused by TNF‐α by inhibiting the NF‐κB pathway.^111^ Recently, IL‐38, a member of the IL‐1 cytokine subfamily, was shown to exert an anti‐inflammatory function in IDD. IL‐38 is upregulated in both human and rat degenerated IVD. In vitro, IL‐38 inhibits activation of the NF‐κB signalling pathway in an inflammation model based on human NP cells by reducing the expression level of NF‐κBp65 protein, and significantly reduces IL‐1β induced by TNF‐α in the NP. IL‐6, COX‐2, MMP‐13 and ADAMTS‐5 expression. IL‐38 also reduces TNF‐α expression leading to decreases in levels of type II collagen and aggrecan.^112^ Therefore, these inhibitory factors may be useful for IDD prevention and treatment by overcoming NF‐κB pathway activation (Figure 2).

## 6 EPIGENETICS ASSOCIATED WITH NF‐κB IN IDD

### 6.1 Histone deacetylases (HDACs)

Epigenetic changes of histones and non‐histones play crucial roles in the process of IDD. In fact, HDACs seem to affect NF‐κB activity and catabolic gene expression in NP cells. Interestingly, inhibition of NAD‐dependent deacetylases (Class III) was reported to show a beneficial effect on IDD.^4^ Sirtuins are a class of evolutionarily conserved nicotinamide adenine dinucleotide (NAD+)‐dependent histone deacetylases and are the main molecules that mediate life extension or delay ageing‐related diseases.^113^ To date, seven members of the sirtuin family have been identified, SIRT1‐SIRT7, which belong to the class III HDACs and share a common catalytic core domain. SIRT1 can directly deacetylate p65 at residue 310 of lysine, thereby inhibiting the transactivation ability of p65 and inhibiting NF‐κB‐dependent gene transcription.^114^ Previous studies have shown that downregulating the level of SIRT1 in NP cells increased NF‐κBp65 and p‐NF‐κBp65 levels. This transcription factor leads to increased levels of various inflammatory factors (TNF‐α, IL‐1β and IL‐6), decreases in type II collagen and aggrecan levels and increases MMP‐13 and ADAMTS‐5 levels, thereby inducing NP cell anabolic metabolism. Significantly, the imbalance between catabolic activities initiates or exacerbates IDD.^115^ SIRT6 at the chromatin level attenuates NF‐κB signalling by deacetylating histone H3K9. The physical interaction between SIRT6 and NF‐κB catalytic subunit p65 affects its transcriptional activity and thus affects the metabolism of NP ECM.^116^, ^117^ The above results indicate that SIRT1 and SIRT6 have protective roles in maintaining the structure and function of the NP. Supporting this concept, SIRT2, the closest homolog of SIRT1, can enable deacetylation of p65 Lys310 to regulate the expression of NF‐κB‐dependent genes,^118^ and SIRT5 and SIRT7 regulate NF‐κB activity by promoting the acetylation of p65.^119^, ^120^ SIRT3 and SIRT4 are also involved in the regulation of NF‐κB.^121^, ^122^ Therefore, we speculate that SIRT2, 3, 4, 5 and 7 may also participate in the development of IDD by regulating the NF‐κB pathway. However, their role in the pathogenesis of IDD is still unclear (Figure 2).

In contrast to the above effects, the classical zinc‐dependent histone deacetylases (class I and II) accelerate destruction of articular cartilage in patients with osteoarthritis (OA) by regulating the activity of NF‐κB.^123^ Treatment with the HDAC6‐specific inhibitor, ACY‐1215, or pan‐HDAC inhibitor, SAHA, inhibited NF‐κB activation and catabolic gene expression in IL‐1β‐stimulated chondrocytes.^124^, ^125^ As NP cells are composed of small chondrocyte‐like cells with typical morphology, they share a common developmental lineage with chondrocytes at the molecular level.^33^ However, the effects of histone deacetylases (class I and II) on NP by regulating the activity of NF‐κB are still unclear. Although further studies are needed to clarify the detailed molecular mechanisms underlying the roles of HDACs in IDD, these studies have shown that the activation of sirtuins has a beneficial effect in IDD. Although further studies are required, we speculate that the inhibition of classical HDACs will also have a beneficial role in IDD treatment.

### 6.2 Non‐coding RNAs

It has recently been reported that IDD is a complex cellular process mediated by multiple molecules, and it is becoming clear that non‐coding RNAs are key factors affecting the pathogenesis of IDD. Several types of non‐coding RNAs have been described, including microRNAs (miRNAs), long non‐coding RNAs (lncRNAs) and circular RNAs (circRNAs). Here, we summarize the non‐coding RNAs related to NF‐κB signalling in NP cells (Table 1 and Figure 2).

**TABLE 1:** Non‐coding RNAs involved in NF‐κB signalling in IDD

Non‐coding RNA(s)	Expression level in IDD	Upstream or downstream of NF‐κB	Target Gene(s)	Function(s) in NP cells	References
miRNA(s)					
miR‐640	Upregulated	Upstream	LRP1	Promote ECM catabolism	^128^
miR‐141	Upregulated	Upstream	SIRT1	Promote ECM catabolism; Promote apoptosis and inhibit proliferation	^115^
miR‐616‐5p	Upregulated	Upstream	Sox9	Promote ECM catabolism; Increase the secretion of inflammatory factors	^140^
miR‐150	Downregulated	Upstream	P2X7	Inhibit ECM catabolism, inflammation and NP cell apoptosis，	^132^
miR‐26a	Downregulated	Upstream	HMGB1	Promote ECM catabolism	^137^
miR‐194	Downregulated	Upstream	TRAF6	Promote ECM catabolism and increase the secretion of inflammatory factors	^129^
miR‐223	Downregulated	Upstream	Irak1	Promote ECM catabolism and increase the secretion of inflammatory factors	^130^
miR‐155	Downregulated	Upstream	TCF7L2	Promote ECM catabolism	^12^
MiR‐202‐3p	Downregulated	Upstream	MMP‐1	Promote ECM catabolism	^131^
miR‐146a	Downregulated	Upstream	ND	Inhibit the secretion of inflammatory factors	^134^
miR‐583	Downregulated	Upstream	BTRC	Promote ECM catabolism	^139^
miR‐27a	Upregulated	Upstream	ND	Promote the secretion of inflammatory factors	^126^
miR‐494	Upregulated	Upstream	SOX9	Promote ECM catabolism; Promote NP cell apoptosis	^127^
miR‐625‐5p	Upregulated	Downstream	ND	Promote NP cell apoptosis	^135^
lncRNAs					
TUG1	Upregulated	Upstream	miR‐26a	Promotes catabolism	^137^
circRNAs					
circ‐4099	Upregulated	Downstream	Sox9	Promote ECM catabolism and increase the secretion of inflammatory factors	^140^
circ‐FAM169A	Upregulated	Upstream	ND	Promote ECM anabolism	^139^

Abbreviations: circRNAs, circular RNAs; IDD, intervertebral disc degeneration; lncRNAs, long non‐coding RNAs; miRNAs, microRNAs; ND, not determined; NP, nucleus pulposus.

miRNA is a type of small non‐coding RNA that can bind to target genes through special sequences to inhibit gene expression. These molecules further aggravate or delay the process of IDD by regulating the NF‐κB signalling pathway. NF‐κB induced by several miRNAs promotes NP degeneration. For example, the expression levels of miR‐141, miR‐27a and miR‐494 are increased in degenerated NP, resulting in acceleration of NP degeneration by activating the NF‐κB signalling pathway. Specifically, miR‐141 knockout was shown to significantly inhibit the expression of MMP‐13, while the expression level of type II collagen was increased in mouse IDD models induced spontaneously or after surgery.^115^ miR‐27a activates NF‐κB signalling and leads to significant increases in the production of proinflammatory factors IL‐1β, IL‐6 and TNF‐α.^126^ miR‐494 promotes ECM degradation and apoptosis of degenerated human NP cells by directly targeting SOX9 to activate NF‐κB signalling.^127^

It is worth noting that the mutual regulation between NF‐κB and miRNA forms a positive feedback loop to further enhance the activity of NF‐κB and accelerate the IDD process; for example, the expression levels of inflammatory factors TNF‐α and IL‐1β mRNA are increased in the degenerated NP tissue, and this inflammatory environment promotes miR‐640 expression through the NF‐κB signalling pathway. miR‐640 in turn targets LRP1 and enhances NF‐κB signalling activity, thereby establishing a positive feedback loop, leading to increased levels of inflammatory factors in the NP microenvironment and accelerating NP degeneration further.^128^ In contrast, many miRNAs have decreased expression levels in degenerated NPs, such as miR‐194, miR‐223, miR‐202‐3p, miR‐150, miR‐155, miR‐202‐3p and miR‐146a. These molecules affect the catabolism of NP cells by activating or inhibiting matrix‐degrading enzymes or molecular components of the NF‐κB signalling pathway. In LPS‐induced degeneration of NP cells, the expression of miR‐194 is reduced. The overexpression of miR‐194 activates the NF‐κB signalling pathway through targeted regulation of TRAF6, which further promotes the expression of inflammatory factors, enhances ECM catabolism and reduces anabolism.^129^ Specifically, miR‐223 overexpression can inhibit the NF‐κB signalling pathway by targeting Irak1 and ultimately inhibit LPS‐induced NP cell inflammation.^130^ The expression of miR‐202‐3p is reduced in severely degenerated human NP and in human NP cells stimulated by IL‐1β. IL‐1β may inhibit the expression of miR‐202‐3p through the activation of NF‐κB in human NP cells, which may further increase the degradation of collagenase II by binding to the target gene, MMP‐1.^131^ miR‐150 can restrict the NF‐κB signalling pathway by targeting P2X7, thereby inhibiting IL‐1β‐induced matrix catabolism, inflammation and NP cell apoptosis.^132^ The expression of miR‐155 is reduced in degenerative IDD. Overexpression miR‐155 inhibits expression of the target gene, TCF7L2, by inhibiting the activity of the p65/RelA promoter to inhibit NF‐κB signalling and prohibit matrix degradation.^12^ TRAF6 is involved in signal transduction of the TNF receptor (TNFR) and IL‐1 receptor/Toll‐like receptor (IL‐1R/TLR) superfamily. It triggers IκB kinase, which in turn triggers downstream NF‐κB transcription factors, and leads to overexpression of proinflammatory cytokines.^133^ miR‐146a reduces the levels of proinflammatory cytokines in NP cells stimulated by LPS by targeting TRAF6 to inhibit the NF‐κB pathway.^134^ In addition, activation of the NF‐κB signalling pathway upregulates miRNAs expression and affects ECM metabolism and cell survival. For example, activation of the TLR4/NF‐κB signalling pathway induces proinflammatory cytokines, further upregulating the expression of miR‐625‐5p, and combines the three main untranslated regions (3′‐UTR) of type I collagen α1 (COL1A1) and thereby leads to the downregulation of COL1A1 and ultimately contributes to cell apoptosis.^135^

The lncRNAs are endogenous non‐coding transcripts longer than 200 nucleotides, and there is accumulating evidence that lncRNAs play key regulatory roles in biological processes. Abnormal lncRNA expression has been shown to be related to a variety of human diseases, including IDD and osteoarthritis.^136^ The role of lncRNA in IDD has recently attracted widespread attention. Tang et al^137^ reported that TUG1 was significantly upregulated in human degenerated NP tissue. TUG1 activates the miR‐26a/HMGB1 axis through the NF‐κB signalling pathway to inhibit the proliferation of NP cells, promote cell apoptosis and accelerate the degradation of extracellular matrix.

The circRNAs, a novel type of widely expressed ncRNA, are different from linear RNAs (miRNAs and lncRNAs) and represent an understudied class of ncRNAs.^138^ As post‐transcriptional regulators, circRNAs interact with miRNAs through competitive endogenous RNA in miRNA sponge and the cytoplasm. miRNA sponge is a circRNA with miRNA binding ability, which can absorb miRNA and inhibit its inhibitory effects on the target. Multiple circRNAs have multiple functions in IDD. Guo et al^139^ reported that the circular RNA, FAM169A, acts as a competitive endogenous RNA and, by targeting miR‐583 and BTRC signals as well as through the NF‐κB signalling pathway, it regulates the imbalance between NP cell ECM anabolism and catabolism, thus affecting the IDD process. The expression of circ‐4099 is increased in degenerated NP tissues, and TNF‐α was shown to upregulate the expression of circ‐4099 in NP cells through the NF‐κB signalling pathway in a dose‐ and time‐dependent manner. It was further demonstrated that circ‐4099 can act as a ‘sponge’ by competitively binding miR‐616‐5p and, by binding to the target gene, Sox9, to regulate the expression of proinflammatory factors in NP cells, including IL‐1β, TNF‐α, IL‐ 6 and PGE2.^140^

## 7 CONCLUSION AND FUTURE PERSPECTIVES

In this review, we summarized the results of published studies on the regulation of the NF‐κB signalling pathway in NP cells during IDD. In the IDD process, many factors (such as inflammation, oxidative stress, mitochondrial dysfunction, mechanical load and nutrient deficiency) directly or indirectly induce matrix‐degrading enzymes (such as MMPs and ADAMTSs) by activating the NF‐κB signalling pathway, thereby accelerating the catabolism level of the NP ECM. Activation of the NF‐κB signalling pathway can increase the expression levels of many inflammatory mediators and chemokines, forming a vicious circle and further accelerating IDD progression. Additionally, the upstream or downstream molecules of NF‐κB, an abnormal expression of NF‐κB subunit binding protein, and epigenetic changes can also affect the survival status of NP cells by regulating the NF‐κB signalling pathway. Therefore, the interaction between NF‐κB and newly identified genes or signalling pathways may reveal many potential therapeutic targets to slow down or reverse the progression of IDD. Although there is a basic understanding of the relationship between the NF‐κB signalling pathway and IDD, many questions in the current knowledge about the mechanism of the NF‐κB signalling pathway in the occurrence and development of IDD remain to be answered. Firstly, it is widely accepted that the non‐canonical NF‐κB signalling pathway is causally related to the progression of various human diseases, including chronic inflammation, autoimmune diseases and malignant tumours.^141^ However, the role of non‐canonical NF‐κB signalling in the NP cells is currently unclear. Therefore, further studies are needed. Secondly, many previous studies have researched the relationship between the NF‐κB signalling pathway and IDD at the cellular and animal levels, with there being a need for further clinical translational research to be carried out. Finally, in the IDD process, the relative importance of the different types of ncRNAs (ie miRNAs, lncRNAs and circRNAs) and their regulatory relationships are not clear. Pleasingly, the studies on the relationship between ncRNA and the NF‐κB signalling pathway in IDD are increasing. Undoubtedly, the research on this relationship is related to the mechanism of IDD development and the provision of new drug targets for IDD treatment which has extremely important clinical significance. Overall, this review summarizes the role of NF‐κB in NP in the IDD process. More and more researches in this area are still needed in the future to further clarify the underlying mechanisms, which will lay a theoretical foundation for the transformation and application of the corresponding molecular targets, and we hope that further research regarding NF‐κB in IDD will yield important discoveries and lead to translational research in the future.

## CONFLICT OF INTEREST

The authors declare no competing interests.

## AUTHOR CONTRIBUTIONS

ZGZ, KXW, LMQ and CHW conceived and wrote the article. ZGZ and WZL contributed to making of figures. ZGZ and KXW wrote the manuscript. ZGZ, GYC, MZJ and HXG revised the manuscript. LMQ and WZLcontributed to proofreading of the article. All authors read and approved the final manuscript.

## Data Availability

Data sharing not applicable to this article as no data sets were generated or analysed during the current study.
